# LDH-TiO_2_ Composite for Selenocyanate (SeCN^−^) Photocatalytic Degradation: Characterization, Treatment Efficiency, Reaction Intermediates and Modeling

**DOI:** 10.3390/nano12122035

**Published:** 2022-06-14

**Authors:** Minaam Hussaini, Muhammad Vohra

**Affiliations:** 1 Civil and Environmental Engineering Department, King Fahd University of Petroleum & Minerals (KFUPM), Dhahran 31261, Saudi Arabia; g201705690@kfupm.edu.sa; 2 Interdisciplinary Research Center for Construction and Building Materials (IRC-CBM), King Fahd University of Petroleum & Minerals (KFUPM), Dhahran 31261, Saudi Arabia

**Keywords:** selenocyanate, layered double hydroxide (LDH), TiO_2_, photocatalysis, RSM

## Abstract

Selenium as a nutrient has a narrow margin between safe and toxic limits. Hence, wastewater discharges from selenium-containing sources require appropriate treatment that considers health concerns and stringent selenium-related water treatment standards. This work examined the use of a photocatalysis-cum-adsorption system based on a layered double hydroxide coupled with TiO_2_ (LDH-TiO_2_) to remove aqueous phase selenocyanate (SeCN^−^), which is difficult to treat and requires specific treatment procedures. The synthesized LDH and LDH-TiO_2_ composite samples were characterized using the X-ray diffraction (XRD), field emission scanning electron microscopy (FESEM), and thermogravimetry analysis (TGA) methods. The XRD results for the uncalcined LDH indicated a hydrotalcite mass with a rhombohedral structure, whereas increasing the calcination temperature indicated transition to an amorphous state. FESEM results for the LDH-TiO_2_ matrix indicated round titanium dioxide particles and LDH hexagonal layers. The TGA findings for uncalcined LDH showed a gradual decrease in weight up to 250 °C, followed by a short plateau and then a sharp decrease in LDH weight from 320 °C, with a net weight loss around 47%. Based on the characterization and initial selenocyanate adsorption results, the 250 °C calcined LDH-TiO_2_ matrix was used for the selenocyanate photocatalysis. A ~100% selenium removal was observed using LDH:TiO_2_ at a 1.5:1 *w*/*w* ratio with a 2 g/L dose, whereas up to 80% selenium removal was noted for LDH:TiO_2_ at a 0.5:1 *w*/*w* ratio. The respective difference in the efficiency of selenium treatment was attributed to enhanced LDH-based adsorption sites in the enhanced LDH:TiO_2_ *w/w* ratio. Furthermore, the selenite and selenate that occurred during SeCN^−^ photocatalytic degradation (PCD) were also nearly completely removed via adsorption. An optimization exercise using response surface methodology (RSM) for total selenium removal showed R^2^ values of more than 0.95, with a prediction accuracy of more than 90%. In summary, the present findings show that the use of a photocatalysis-cum-adsorption system based on LDH-TiO_2_ is a promising technique to treat industrial wastewater discharges for selenocyanate and also remove the resulting intermediates.

## 1. Introduction

Ever-growing water pollution is a serious concern requiring appropriate environmental protection plans [1,2]. To this end, selenium-based water pollution is also a growing concern that requires innovative solutions because selenium as a nutrient has a narrow margin between safe and toxic limits [3]. Some significant selenium discharge sources include major hydrocarbon use and processing facilities, natural geo-resources extraction sites, refineries, the pigment industry, semiconductor manufacturing, and glass production [4,5,6]. Wastewater discharges from such sources require an appropriate treatment that considers health concerns and stringent selenium-related water treatment standards. For example, the USEPA standard mandates the maximum limit of 50 ppb for selenium in natural water supplies [7]. Hence, different technologies have been employed for selenium removal, including membrane modules [8], biosorption [9], adsorption [10], phytoremediation [6], electrocoagulation [11], chemical reduction [12], coagulation and flocculation [13], and ion exchange [14]. However, because of various operational issues, better methodologies for the treatment of selenium-contaminated water bodies are needed. Furthermore, aqueous phase selenocyanate (SeCN^−^), found in several industrial effluents, is typically difficult to treat and requires specific treatment procedures. Some specific selenocyanate sources include mining facilities, large fossil fuel-based setups, and petroleum refineries [4,5,6].

The use of layered double hydroxides (LDHs) has been reported for several environmental pollutant treatments, including arsenic [15], heavy metals [16,17,18], organic dyes [19], radionuclides [20], organic anions [21], and inorganic anions [22]. Among the various processes used for treating wastewaters, the use of photocatalysis offers a green and simple technology [23,24,25] that has been successfully employed for the degradation of various toxic compounds [26,27]. Furthermore, TiO_2_-initiated photocatalytic degradation (PCD) systems are also reported to be efficient for aquatic pollution control [28,29,30,31,32]. Interestingly, efficient performance of an LDH-TiO_2_ matrix has also been demonstrated for aqueous pollution control. Seftel et al. (2010) [33] report higher photocatalytic activity of the LDH-TiO_2_ matrix as compared to that of TiO_2_ alone for the photocatalytic removal of methylene blue. Carja et al. (2010) [21] report the successful application of TiO_2_/ZnLDH for treating aqueous phenol. Furthermore, Paredes et al. (2011) [34] report that a TiO_2_/LDH matrix produced a synergistic effect causing both higher ∙OH radical production and higher degradation of phenol than TiO_2_ alone. Similarly, the application of an LDH-TiO_2_ matrix for dimethyl phthalate and methylene blue pollutant removal has also been reported [35,36], as well as the use of an LDH-TiO_2_ matrix for the removal of 2,4-dichlorophenoxyacetic acid and orange II [37,38]. This clearly indicates improvement in both adsorption and photocatalysis of several aqueous pollutants when using a combined LDH-TiO_2_ system. Furthermore, though the use of LDH for selenite and selenate treatment is reported [7,39,40,41,42], to the best of our knowledge, there has been no study reporting the use of an LDH-TiO_2_ matrix for the removal of selenocyanate (SeCN^−^) and associated selenite/selenate oxyanions. In addition, as mentioned earlier, aqueous phase selenocyanate (SeCN^−^) found in several industrial effluents is typically difficult to treat and requires specific treatment procedures. Hence, considering the recalcitrant nature of selenocyanate and the respective treatment challenges [5], the present work investigates the application of the combined LDH-TiO_2_ matrix for selenocyanate removal along with the effect of different operational variables on process efficiency. This application offers a unique solution where the photocatalysis oxidizes selenocyanate to selenite and selenate followed by their uptake by the LDH-TiO_2_ matrix. Thus, the combined “photocatalysis-cum-adsorption” system offers a two-in-one solution. This study will also explore the possible reaction intermediates along with process optimization using response surface methodology (RSM)-based modeling.

## 2. Materials and Methods

### 2.1. Materials

High-purity chemicals used were aluminum nitrate nonahydrate (Sigma Aldrich, Stuttgart, Germany), titanium dioxide (DEGUSSA P25, Stuttgart, Germany), magnesium nitrate hexahydrate (Sigma Aldrich, Stuttgart, Germany), potassium selenocyanate (Aldrich, Burlington, MA, USA), sodium selenite (Aldrich, Burlington, MA, USA), and potassium selenate (Aldrich, Burlington, MA, USA).

### 2.2. Synthesis and Characterization

The MgAl-LDH (hereupon referred to as LDH) was synthesized using a co-precipitation technique. Magnesium nitrate hexahydrate and aluminum nitrate nonahydrate at a molar ratio of 3:1 (M^2+^:M^3+^) were transferred to 50 mL of distilled water and then stirred in an oil bath at 60 °C for about 15 min at 600 rpm, with pH adjusted to 10 ± 0.5 using NaOH (1 M). This was followed by stirring at 900 rpm with a temperature adjustment to 90 °C, and later on refluxing the suspension for 24 h and then aging it for 4 days at 80 °C [43,44]. A subsequent water-and-ethanol-based washing and 1–2 days of drying at 80 °C delivered the desired LDH that was then appropriately stored. In addition, the LDH:TiO_2_ matrix was prepared as reported earlier [45]. A 10 g/L LDH suspension was gradually introduced into a 10 g/L TiO_2_ suspension at LDH:TiO_2_ ratios of 1.5:1, 1:1, and 0.5:1. This was first followed by mixing for 48 h at room temperature and then centrifuging and drying at 80 °C. The resulting LDH:TiO_2_ matrix was then calcined at 250 °C for 5 h and appropriately stored. The synthesized adsorbents were characterized using X-ray diffraction (XRD—mini-X-ray diffraction, Rigaku Miniflex-II, Tokyo, Japan), field emission scanning electron microscopy (FESEM, Tescan Lyra-3, Brno – Kohoutovice, Czech Republic), and thermogravimetry analysis (TGA, Perkin Elmer TGA 4000 analyzer, Waltham, MA, USA). The XRD 2θ analyses were completed at a scanning rate of 3°/min from 5° to 70°. For the FESEM analyses, the respective materials to be analyzed were first coated with gold to make the surfaces conductive. The TGA analyses were completed in a nitrogen atmosphere from 50 to 800 °C at a step rate of 15 °C/min.

### 2.3. Photocatalytic Degradation (PCD) Experiments

**Figure 1:**
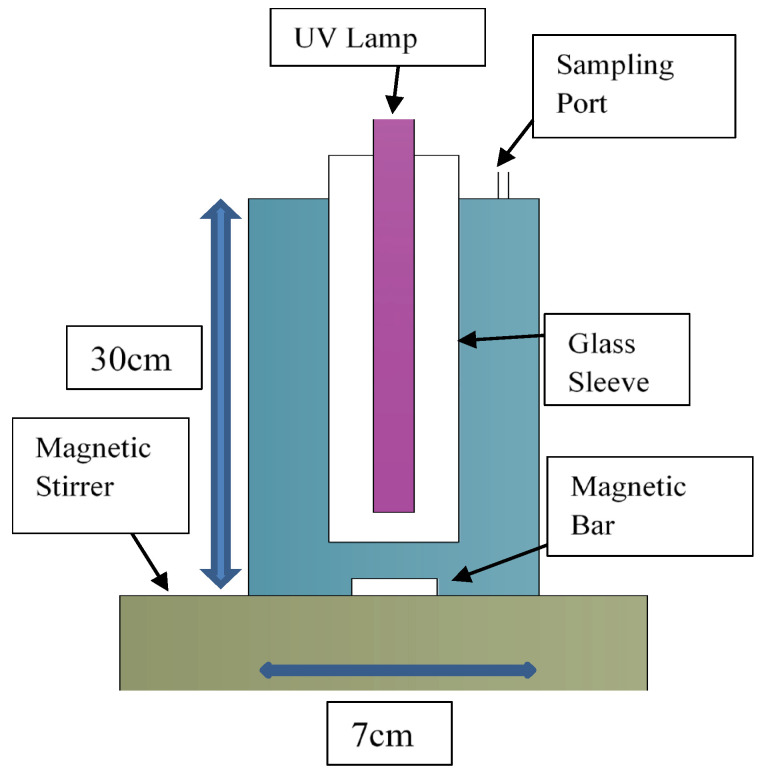
UV reactor setup layout as used for the LDH-TiO_2_ PCD work.

The layout of Pyrex glass reactor used for the PCD studies is given in Figure 1. The shown UV lamp (FT15T8-BLB 15 W, Sankyo Denki, Hiratsuka, Japan) emitted light at 315–400 nm with a peak maximum of ~352 nm. The synthetic wastewater samples for all the experiments were prepared using SeCN^−^ 1000 mg/L standard. A blank sample was always collected for each experiment before adding LDH-TiO_2_, and, then, the photocatalyst/adsorbent was mixed with the remaining synthetic wastewater batch followed by a sample collection at 30 min to assess any initial adsorption. After this, the UV lamp was turned on, with several samples taken till 6 h, which were then tested for the selenium and other ionic species using an advanced ion chromatography setup (Metrohm). The column used for the IC analyses was “Anion Dual 2” and the eluents used were 1.3 mM Na_2_CO_3_ and 2 mM NaHCO_3_.

### 2.4. Response Surface Methodology (RSM)

As shown in the Table 1, a three-level face-centered central composite design (FC-CCD) with a single center point was employed for the response surface methodology (RSM)-based design of experiments for the photocatalysis work. The respective design of experiments is provided in Table 2.

## 3. Results

### 3.1. LDH and LDH-TiO_2_ Matrix Characterization

**Figure 2:**
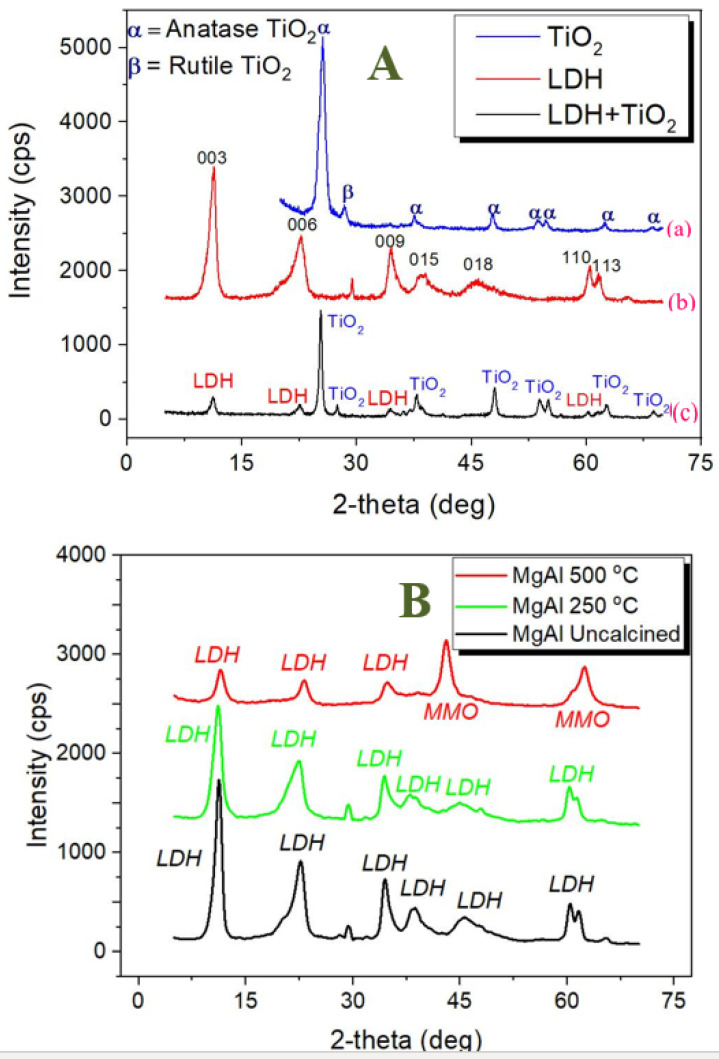
XRD profiles of: (**A**)-(a) Degussa P25 showing anatase and rutile phases, (**A**)-(b) synthesized uncalcined LDH, (**A**)-(c) TiO_2_-modified LDH calcined at 250 °C (L:T ratio = 1:1). (**B**) LDH with the effect of calcination temperature (the synthesis temperatures are in the top-right corner).

Initially, the synthesized LDH and LDH-TiO_2_ matrix samples were characterized using several advanced techniques. The XRD profiles of uncalcined TiO_2_, uncalcined LDH, and calcined LDH-TiO_2_ (250 °C), are shown in Figure 2A. The XRD profile of TiO_2_ (Figure 2A-(a)) shows both anatase and rutile phases as also reported earlier [46]. The presence of both rutile and anatase phases is reported to yield better photocatalysis efficiency [47]. Furthermore, the XRD results for the uncalcined LDH (Figure 2A-(b)) indicate a hydrotalcite mass with a rhombohedral structure (3R poly-type) based on the basal (003, 006, 009, 015, and 018), and non-basal (110 and 113) reflections [48,49]. The corresponding cell parameters i.e., a, c, and d_003_ for uncalcined LDH were found to be 0.307, 2.407 and 0.807 nm, respectively ((c is equal to ((6d_006_ + 3d_003_)/2) and a is equal to 2d_110_). The basal spacing (d_003_) of 0.807 nm indicates nitrate in LDH as revealed by the presence of nitrogen in the EDX spectra analysis (Table 3) [49,50]. Moreover, theses cell values along with the sharp XRD peaks in Figure 2A-(b) for LDH (003, 006, 110, and 113) represent a well-crystallized LDH structure [48]. Furthermore, for the LDH-TiO_2_ matrix, the respective XRD results (Figure 2A-(c)) show both LDH and TiO_2_ peaks that suggests incorporation of TiO_2_ into the LDH phase. On the other hand, the XRD findings for LDH (Figure 2B) show that increasing the calcination temperature decreases the intensity of the peaks, indicating transition to an amorphous state [51,52], and the two new peaks (at 43.1 and 62.7) for the calcined LDH at 500 °C correspond to the periclase (MgO) phase [22].

**Figure 3:**
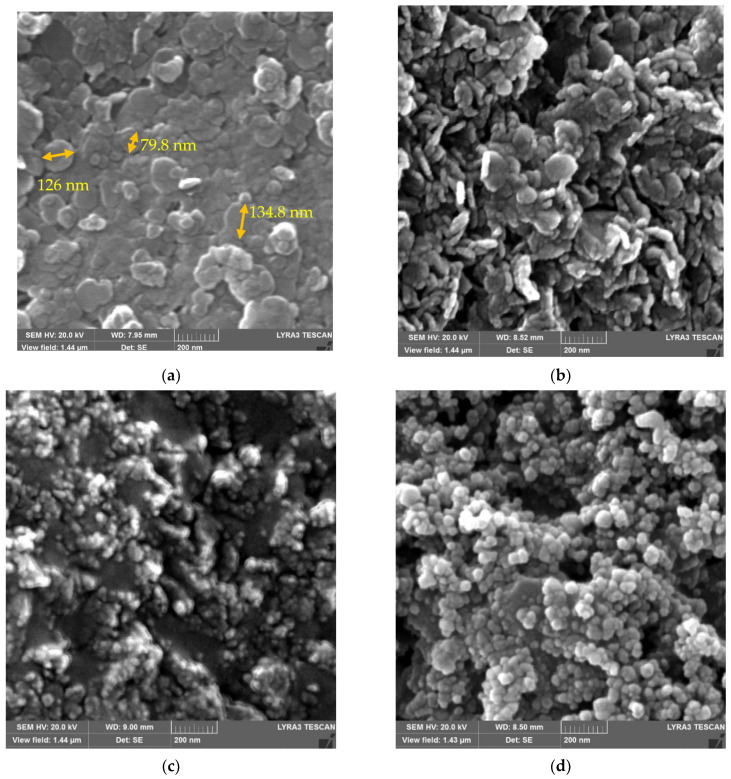

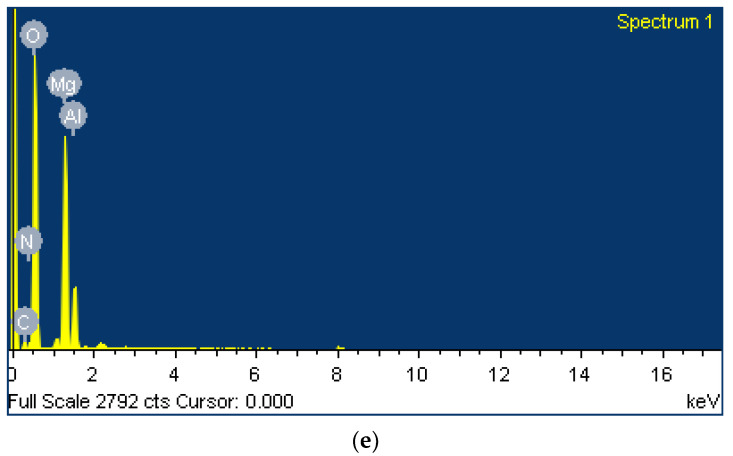
Results from (**a**) FESEM analysis of uncalcined LDH; (**b**) FESEM analysis of LDH calcined at 250 °C; (**c**) FESEM analysis of LDH calcined at 500 °C; (**d**) FESEM analysis of Degussa P25 TiO_2_ nanoparticles; (**e**) EDX analysis of uncalcined LDH.

**Figure 4:**
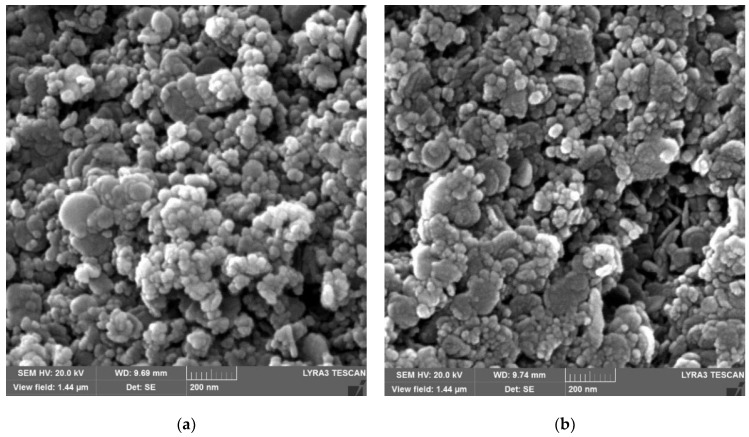

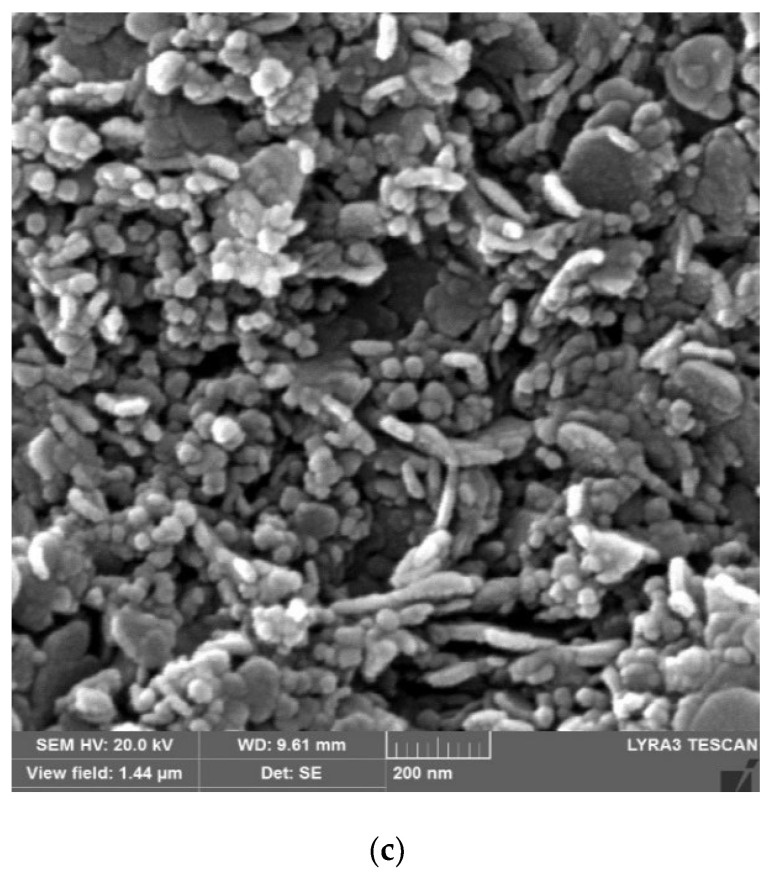
Results from FESEM analysis for LDH-TiO_2_ matrix synthesized at 250 °C at (**a**) 0.5:1, (**b**) 1:1, and (**c**) 1.5:1 ratios.

The aforementioned trends for the LDH are also supported by the FESEM findings as given in Figure 3a–c for several LDH samples. Figure 3a reveals hexagonal LDH crystals in the nm range. Nevertheless, with an increase in calcination temperature to 250 °C (Figure 3b), the hexagonal LDH plates become somewhat rougher, and, at 500 °C (Figure 3c), they disappear. This is in accordance with the respective XRD results (Figure 2B) revealing that an increase in temperature renders the LDH amorphous [51]. Furthermore, the FESEM results in Figure 3e and Table 3 for the uncalcined LDH reveal that the Mg:Al ratio is 3.4, whereas C (carbon) results from the CO_3_^2−^ ion induction in LDH [53]. The morphology of Degussa P25 TiO_2_ nanoparticles with a particle size of approx. 30 nm (Figure 3d) is also in accordance with the literature [54,55], whereas the FESEM results in Figure 4a–c for the calcined LDH-TiO_2_ matrix indicate round titanium dioxide particles and LDH hexagonal layers with the former showing a diminishing trend from “a” to “c”.

**Figure 5:**
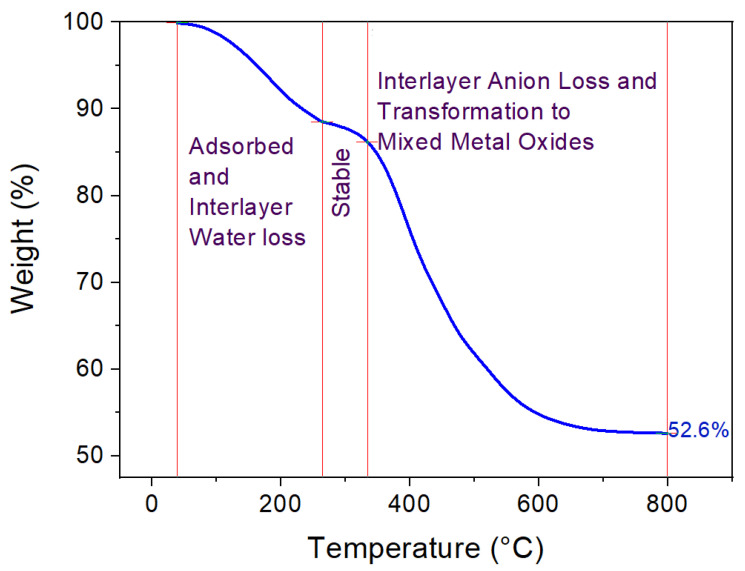
Results from the TGA analysis of uncalcined LDH.

The TGA findings for uncalcined LDH (Figure 5) show a gradual decrease in weight up to 250 °C, indicating the loss of adsorbed and interlayer water. This is followed by a short plateau and then a sharp decrease in LDH weight from 320 °C, which corresponds to the removal of LDH-matrix-bound NO_3_^−^, CO_3_^2−^, and OH^−^ molecules [56,57,58,59]. The net weight loss for the uncalcined sample is around 47%. These findings are qualitatively in accordance with the aforementioned XRD (Figure 2B) and FESEM findings (Figure 3a–c), starting from the uncalcined to calcined LDH samples, where the LDH samples at elevated calcination temperatures were noted to have an amorphous phase state. The above surface characterization findings indicate that a moderate calcination temperature of 250 °C could potentially synthesize a better LDH-TiO_2_ matrix, as also noted from the photocatalysis results for the selenocyanate species reported below.

### 3.2. Selenocyanate Photocatalytic Degradation Using LDH:TiO_2_ Matrix

Initially, a preliminary set of adsorption experiments was completed to evaluate the selenocyanate adsorption capacity of synthesized LDH:TiO_2_ samples, and the 250 °C LDH sample delivered the maximum selenocyanate retention. It was noted that a maximum removal efficiency of only ~40% for SeCN^−^ was achieved using LDH:TiO_2_ calcined at 250 °C without using UV (SeCN^−^ initial concentration = 9 ppm, dose = 1 g/L). Furthermore, it was also noted that using only TiO_2_ without UV had negligible effect on the SeCN^−^ removal. Moreover, using UV with TiO_2_ led to the complete transformation of SeCN^−^ into SeO_4_^2−^ within 6 h of UV irradiation, but no removal of SeO_4_^2−^ occurred. Hence based on this, along with the findings from the surface characterization exercise (Section 3.1), the 250 °C calcined LDH-TiO_2_ matrix was further employed for detailed work on the photocatalytic degradation (PCD) of selenocyanate species. It is also important to note that immediately upon LDH-TiO_2_ sample addition before PCD, the pH increased approximately to 9 as also noted earlier [60,61], which is attributed to the release of hydroxide groups from LDH [62,63,64,65], as summarized in Equations (1) and (2) [42,66]:

(1) $${\mathrm{Mg}}_{\left(1-x\right)}{\mathrm{Al}}_{x}(\mathrm{OH}{)}_{2}({\mathrm{CO}}_{3}{)}_{x/2}\;\to \;{\mathrm{Mg}}_{\left(1-x\right)}{\mathrm{Al}}_{x}O_{\left(1+x/2\right)}+\frac{x}{2}{\mathrm{CO}}_{2}+H_{2}O$$

(2) $${\mathrm{Mg}}_{\left(1-x\right)}{\mathrm{Al}}_{x}O_{\left(1+x/2\right)}+\frac{x}{n}An^{-}+(1+x/2)H_{2}O\;\to \;{\mathrm{Mg}}_{\left(1-x\right)}{\mathrm{Al}}_{x}(\mathrm{OH}{)}_{2}A_{\left(x/n\right)}+{\mathrm{xOH}}^{-}$$

Furthermore, during photocatalysis, because of active species including ∙OH radicals and hole (h^+^) species [32,67], the selenocyanate initially breaks down to selenium and cyanide species; the selenium is then oxidized to selenite and then to selenate (SeCN^−^ →SeO_3_^2−^→SeO_4_^2−^), while CN^−^, due to oxidation carried out by photogenerated holes (h^+^), converts to OCN^−^, as given below in Equations (3)—(5) [67,68]:

(3) $${\mathrm{SeC}N}^{-}+3H_{2}O\overset{{\mathrm{TiO}}_{2}}{\to }{\;\mathrm{SeO}}_{3}^{2-}+\mathrm{HCN}+5H^{+}+4e^{-}$$

(4) $${\mathrm{SeO}}_{3}^{2-}+H_{2}O\overset{{\mathrm{TiO}}_{2}}{\to }{\;\mathrm{SeO}}_{4}^{2-}+2H^{+}+2e^{-}$$

(5) $${\mathrm{CN}}^{-}+2h^{+}+2{\mathrm{OH}}^{-}\underset{\;}{\to }{\;\mathrm{OCN}}^{-}+H_{2}O\;$$

**Figure 6:**
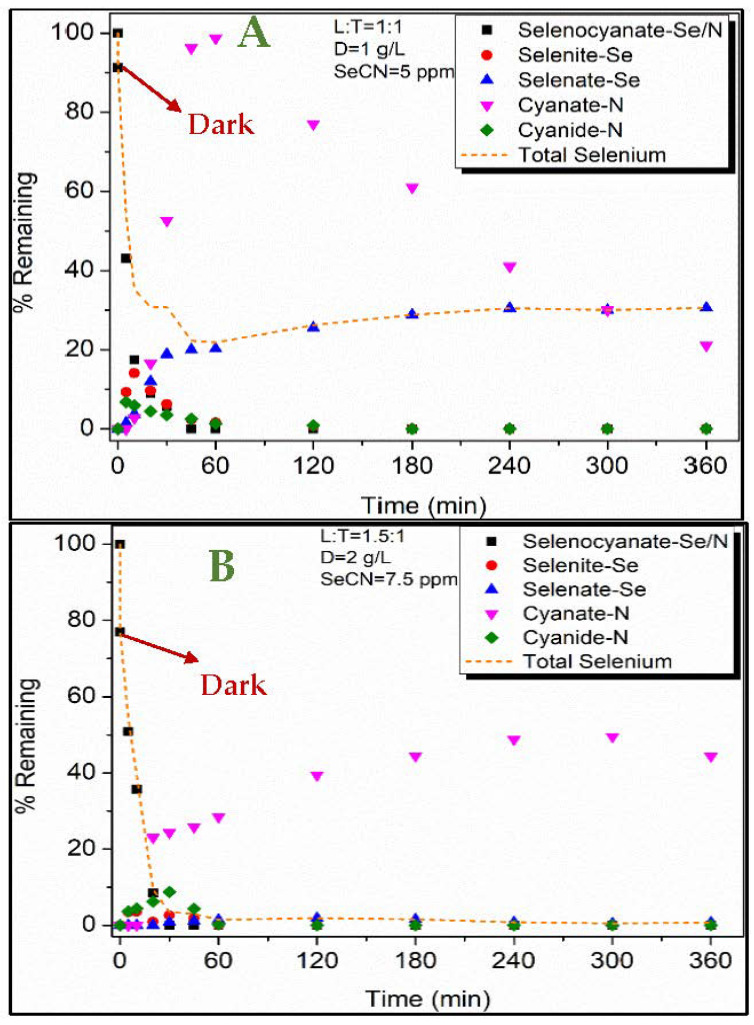

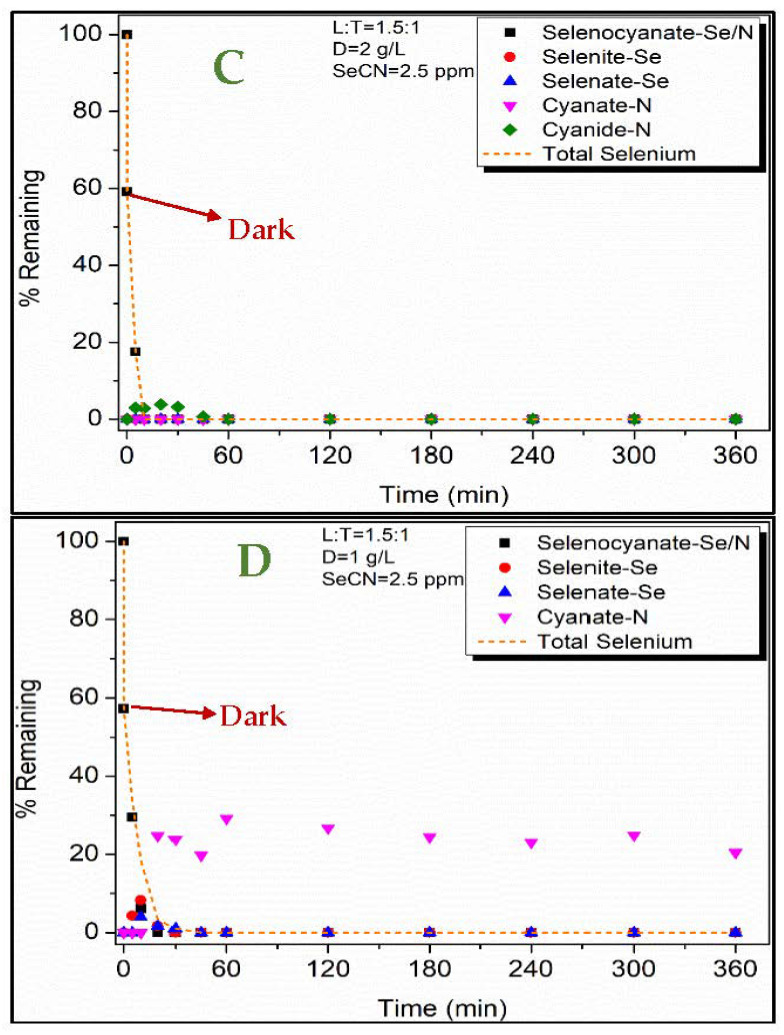

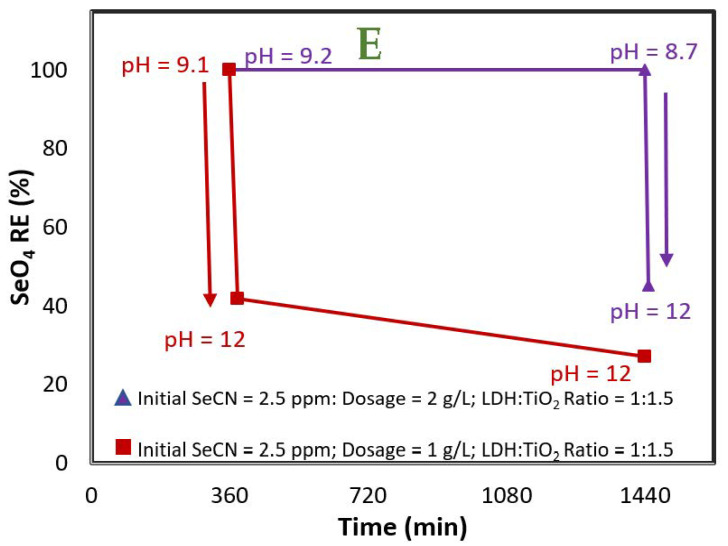
(**A**–**D**) PCD profile of SeCN^−^ under different operating conditions; (**E**) pH influence on percentage of SeO_4_^2−^ adsorption (RE (%)).

These transformations were also noted in the present work (Figure 6A) with the formation of cyanide, cyanate, selenite, and selenate as the reaction by products. The respective OCN^−^ results (Figure 6A) also show a hump-type trend that can be attributed to the uptake of OCN^−^ by the LDH:TiO_2_ matrix. A similar trend is also noted for selenium removal with selenite converting to selenate (Figure 6A). These findings show that the LDH:TiO_2_ matrix effectively removes both selenocyanate and the resulting reaction byproducts.

**Figure 7:**
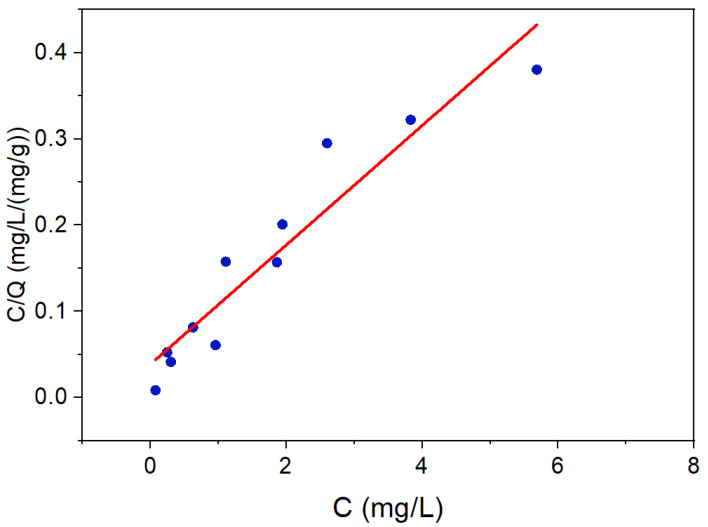
Langmuir isotherm fitting for selenate removal using LDH-TiO_2_ matrix.

Figure 6B,C show no significant presence of aqueous selenite or selenate species. This could have resulted from respective selenium species being adsorbed at the LDH surface. To ascertain this, the pH of system in Figure 6C (after 6 h photocatalysis) was increased to 12 to cause the release of adsorbed selenium species. As shown in Figure 6E, this resulted in the release of adsorbed selenate species (the red line in Figure 6E), as also noted in other LDH-based studies [41,69]. This could be explained by LDH’s pH_zpc_ ~9 and the resulting electrostatic repulsion occurring between the anionic selenate species and adsorbent surface sites [70]. Nevertheless, these findings confirm the oxidation of SeCN^−^ first to selenite and then to selenate. A similar trend was also noted for the system shown in Figure 6D, wherein significant SeO_4_^2−^ of approx. 76% desorbed in the aqueous phase (the violet line in Figure 6E). These results thus confirm the uptake of SeO_4_^2−^ by the adsorbent matrix. The adsorption of selenate (onto the LDH-TiO_2_ matrix) was also noted to fit to the classical Langmuir model (Figure 7; compared to the Freundlich model), as per Equation (6):

(6) $$\frac{C_{eq}}{Q_{eq}}=\frac{1}{Q_{m}b}+\frac{C_{eq}}{Q_{m}}$$

where *C_eq_* (mg/L), *Q_eq_* (mg/g), *Q_m_* (mg/g), and *b* (L/mg) are the standard Langmuir isotherm parameters. The *Q_m_* for selenate was 14 mg/g, with similar values also reported earlier (Constantino et al., 2017 and Tian et al., 2017), indicating monolayer coverage for selenate adsorption onto LDH (Paikaray et al., 2013). The respective values of the slope, intercept, *b*, and adjusted R^2^ were found to be 0.07, 0.04, 1.8 L/mg, and 0.8946, respectively.

The above findings show that the LDH-TiO_2_ matrix can remove both the selenocyanate complex and the resulting selenium species during the combined photocatalytic treatment.

### 3.3. RSM Modeling of Photocatalytic Degradation Process

The present work was expanded to further realize the effect of respective operational variables on selenium removal efficiency utilizing the response surface methodology (RSM)-based experimental design approach (Table 2). To this end, initially two RSM models, namely, the residual selenate model (RS; Equation (7)) and total selenium removal model (TS; Equation (8)), were developed (based on the results from Table 2) for predicting the remaining selenium after selenocyanate photocatalysis. The respective results as given in (Table 4) show that the reduced quadratic equation yields a good model for SeO_4_^2-^ residual, whereas for total selenium removal, the logit-transformed full quadratic model showed the best results. For the RS model (Equation (7)), the significance of the model terms with *p*-values < 0.05 shows that the respective terms significantly contribute toward improving the model results [68]; however, for the TS model (Equation (8)), only the term B^2^ has a *p*-value slightly higher than 0.05.

**Figure 8:**
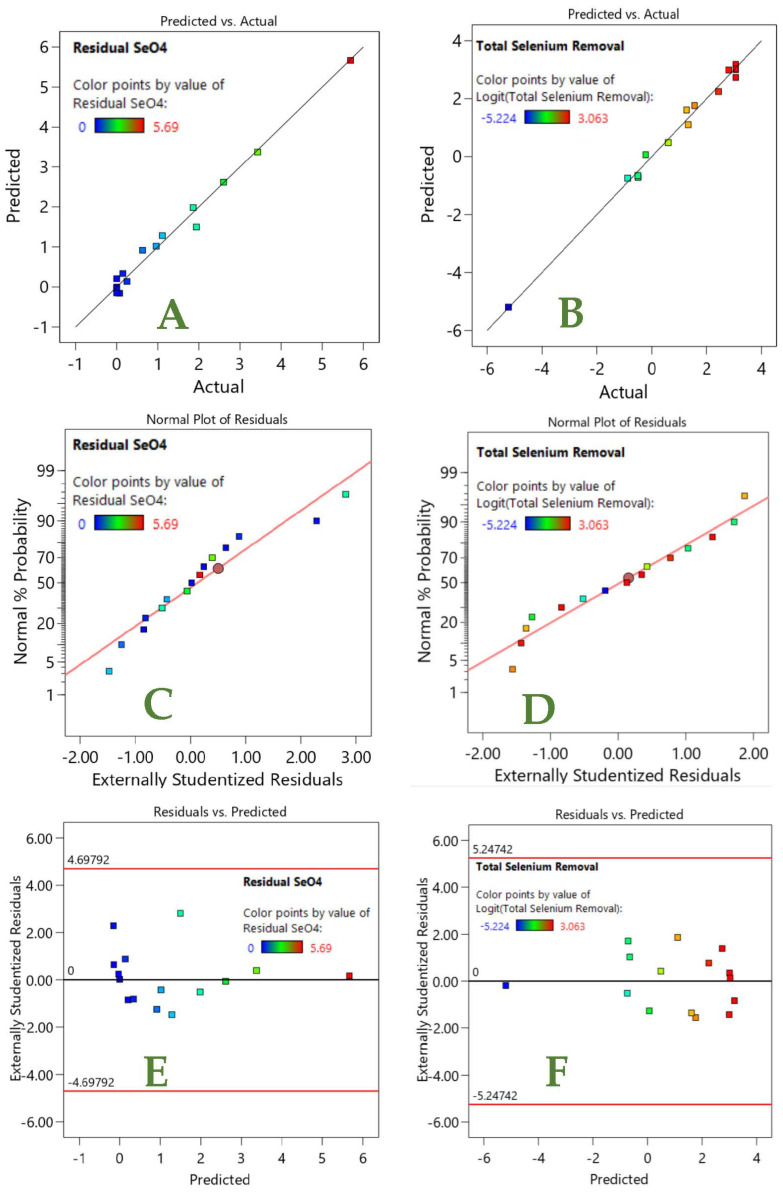
Predicted vs. actual plots for (**A**) RS and (**B**) TS models; normal plot of residuals for (**C**) RS and (**D**) TS models; residual vs. predicted plot for (**E**) RS and (**F**) TS models.

Both the RS (Equation (7)) and TS (Equation (8)) models show high R^2^ values (Table 5), and the differences between the adjusted R^2^ and predicted R^2^ values is less than 0.2, which is also indicative of good prediction accuracy, as shown in Figure 8A,B. This suggests that the model predictions closely follow the experimental values. Furthermore, the adequate precision values (ratio of signal to noise) of 29.99 and 34.46 for the RS and TS models, respectively, which are >4, are also indicative of good model fit.

(7) $$Residual\;{\mathrm{SeO}}_{4}^{2-}=1.506-4.6775A-1.13B+1.4425C+1.115AB-\;0.673AC-0.229BC+1.98A^{2}$$

(8) $$\begin{array}{ll} Logit(TSR)= & Ln\left[\frac{{\mathrm{RE}\;\mathrm{SeO}}_{4}-46.5}{102.5-{\mathrm{RE}\;\mathrm{SeO}}_{4}}\right]\\ & =-7.639+9.09742A+8.24054B-1.55023C-2.65370AB\\ & +0.480254AC+0.413407BC-1.87331A^{2}-1.82388B^{2} \end{array}$$

where
*A*=LDH:TiO2 ratio (0.5:1.5);*B*=adsorbent dosage (1:2 g/L);*C*=selenocyanate concentration (2.5 to 7.5 mg/L);*Residual* SeO_4_^2−^=residual concentration of selenate in solution after 6 h of UV irradiation (mg/L);*TSR*=selenocyanate removal efficiency expressed as total selenium removed (%).

The other statistical factors, including the normal plot of residuals (Figure 8C,D) and the residual vs. predicted results (Figure 8E,F), show randomness with no specific pattern, providing additional model suitability information. For both the RS and TS results, the respective outcomes show that the assumptions of normality (Figure 8C,D) and randomness (Figure 8E,F) are valid, which further supports the proposed RS and TS models.

**Figure 9:**
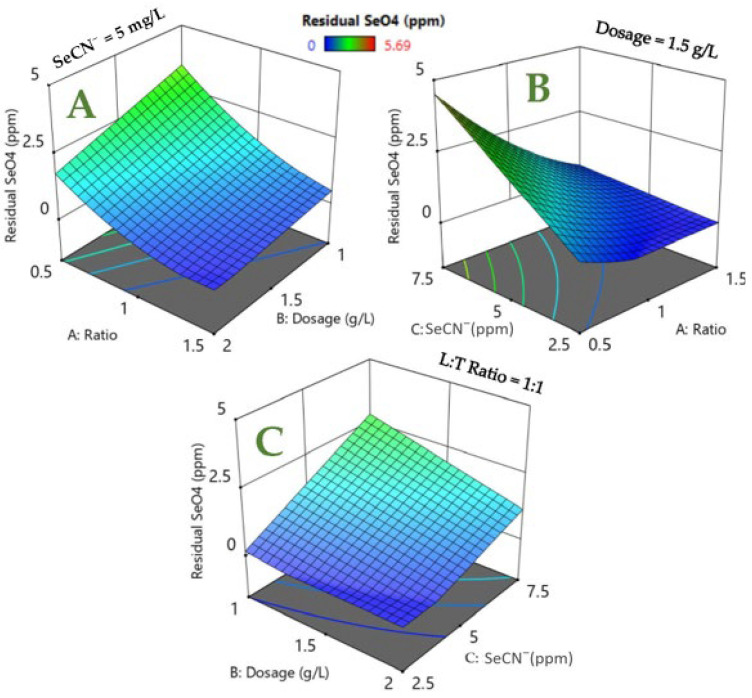

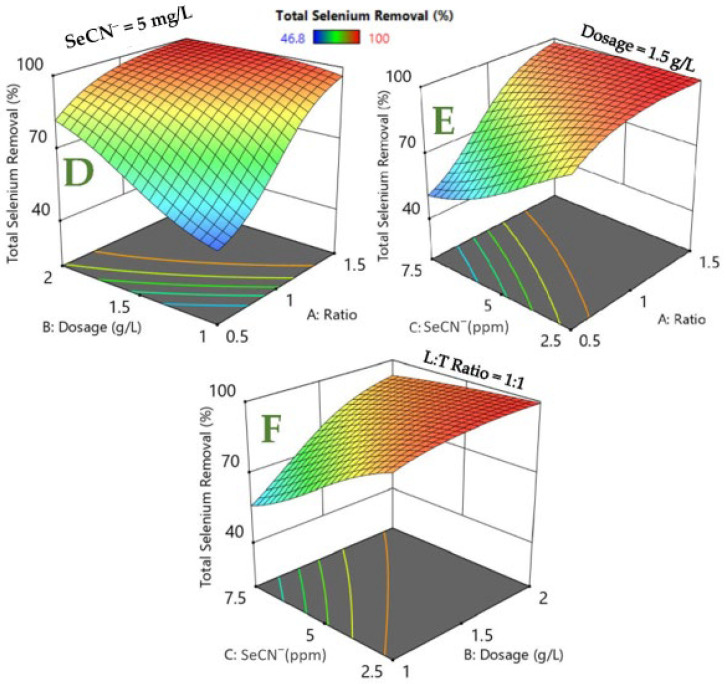
RSM profiles: (**A**) residual SeO_4_^2−^ (initial SeCN^−^ = 5 mg/L); (**B**) residual SeO_4_^2−^; (**C**) residual SeO_4_^2−^. Total selenium removal (%) with; (**D**) total selenium removal; (**E**) total selenium removal; (**F**) total selenium removal.

The selenium removal results from the respective RSM studies are summarized in Figure 9. In general, for SeCN^−^ 5 mg/L, enhanced LDH:TiO_2_ (L:T) initiates enhanced SeO_4_^2−^ removal with L:T 1.5 at 2 g/L delivering near-complete SeO_4_^2−^ removal (Figure 9A,D). The respective increase in SeO_4_^2−^ treatment efficiency can be attributed to enhanced LDH-based sites at an enhanced LDH:TiO_2_ (L:T) ratio. This is further corroborated by the fact that the variation in residual selenate and total selenium removal with the adsorbent dosage is reduced at the LDH:TiO_2_ 1.5 ratio rather than at 0.5 (Figure 9A,D). In general, near-complete selenium removal (~100%, ~0 mg/L residual selenate) can be observed at optimum process conditions. Furthermore, starting with selenocyanate at 5 mg/L (Figure 9D), the total selenium removal varies from 47 to 80% for the LDH:TiO_2_ 0.5 ratio, whereas for the LDH:TiO_2_ ratio of 1.5, approx. 95% near-complete treatment is noted. Furthermore, the remaining SeO_4_^2−^ decreases with a decrease in initial selenocyanate, yielding higher selenium removal (Figure 9B,C,E,F). Qualitatively similar observations were made using 1.5 g/L adsorbent at initial SeCN^−^ measurements of 2.5 and 7.5 mg/L. In summary, the variations in the residual selenate and total selenium removal are prominent at a reduced LDH:TiO_2_ ratio and reduced dose, indicating the need for a high LDH:TiO_2_ ratio and dose for a higher overall selenium removal efficiency.

### 3.4. Optimization of the Photocatalytic Degradation Process

Finally, an optimization study was performed with the aim of maximizing removal while minimizing the LDH-TiO_2_ dosage for the highest SeCN^−^ concentration considered in this study, i.e., 7.5 mg/L. Table 6 provides the factor constraints employed in the current optimization study, with the desirability (i.e., nearness of a response to the ideal quantity) between zero and one, i.e., lowest to highest [71]. For the optimization process, the lower and upper weights were kept as 1, and the importance value was set to 3, which are the default values for the optimization process.

The PCD model optimization process generated 39 solutions for the stated objective function, with the desirability varying from 0.890 to 0.776, out of which the optimum removal for 7.5 mg/L SeCN^−^ that could be achieved by employing minimum LDH-TiO_2_ dosage of 1 g/L was ~89% using an LDH-TiO_2_ ratio of 1.5:1. Thus, the LDH-TiO_2_ matrix displays high removal efficiencies even at lower dosages (for LDH-TiO_2_ 1.5:1).

**Figure 10:**
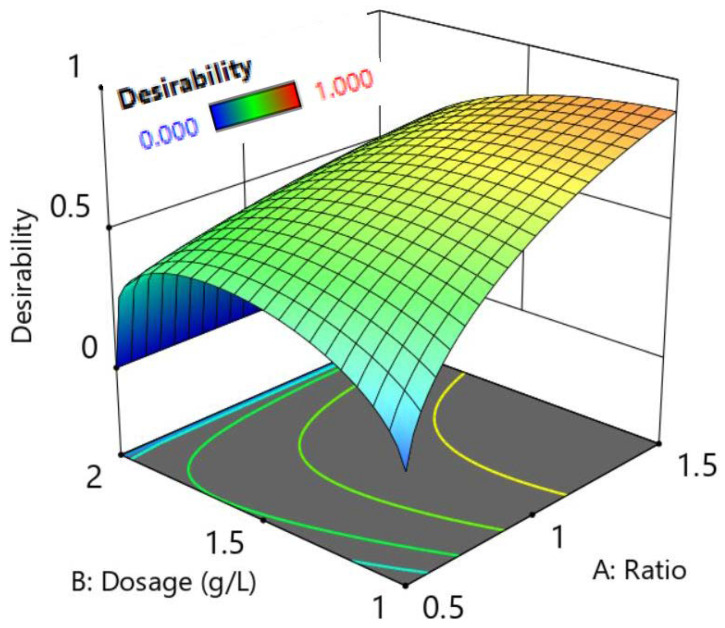
Desirability function variation for PCD optimization process (SeCN^−^ = 7.5 mg/L).

The desirability function for LDH-TiO_2_ 0.5:1 displays a parabolic variation (Figure 10) owing to the objectives that were defined in this study, i.e., maximizing the removal of pollutants and minimizing the dosage of the adsorbent. However, an increase in the dosage increases the selenium removal, albeit leading to lower desirability and thus resulting in a parabolic variation. The variation at a higher LDH-TiO_2_ ratio of 1.5:1 shows a peak at a dose value of 1 g/L and is indicative of the efficient performance of LDH-TiO_2_ at a ratio of 1.5:1, even at lower dosage values. For SeCN^−^ 7.5 mg/L and employing minimum an LDH-TiO_2_ dosage of 1 g/L, the maximum selenate removal of ~89% can be achieved by using an LDH-TiO_2_ ratio of 1.5:1. Thus, LDH-TiO_2_ matrix displays high removal efficiencies even at lower dosages (for an LDH-TiO_2_ ratio 1.5:1).

## 4. Conclusions

The present study employed the LDH-TiO_2_ matrix for aqueous phase selenocyanate (SeCN^−^) treatment using photocatalytic degradation (PCD) and also extended the characterization results for synthesized LDH and LDH-TiO_2_ samples along with response surface methodology (RSM)-based modeling findings. The XRD results for the uncalcined LDH indicate a hydrotalcite mass with a rhombohedral structure, whereas the FESEM results for the LDH-TiO_2_ matrix indicate round titanium dioxide particles and LDH hexagonal layers. Based on the characterization and initial selenocyanate adsorption results, the 250 °C calcined LDH-TiO_2_ matrix was used for selenocyanate photocatalytic degradation. A ~100% selenium removal was observed using an LDH:TiO_2_ ratio of 1.5:1 with 2 g/L dose, whereas up to 80% selenium removal was noted for the LDH:TiO_2_ at a ratio of 0.5:1. Furthermore, the selenite and selenate that occurred during SeCN^−^ photocatalytic degradation (PCD) were also almost completely removed via adsorption. In addition, both the residual selenate (RS) and total selenium removal (TS) RSM models yielded high R^2^ values, and the differences between the adjusted R^2^ and predicted R^2^ values were less than 0.2, which is also indicative of good prediction accuracy. This suggests that the respective RSM model predictions closely follow the experimental values.

## Figures and Tables

**Table 1:** Levels and factors for photocatalysis experiments using RSM modeling.

Factors	Level −1	Level 0	Level 1
A (*w*/*w* ratio of LDH:TiO_2_ matrix)	0.5	1	1.5
B (dosage of LDH:TiO_2_ matrix (g/L))	1.0	1.50	2.0
C (selenocyanate (mg/L))	2.50	5.0	7.50

**Table 2:** The RSM based design of experiments for the photocatalysis work.

Exp No.	LDH:TiO_2_ Ratio	Dosage (g/L)	SeCN^−^ (mg/L)	Residual SeO_4_^2−^ (mg/L)	Total Selenium Removal (%)
1	1	1.5	5	0.63	90.2
2	1	2	5	0.15	98
3	1	1	5	1.94	71.4
4	1	1.5	7.5	1.86	82.7
5	1.5	2	7.5	0.07	99.3
6	1.5	2	2.5	0	~100
7	0.5	1.5	5	2.6	63
8	1.5	1	7.5	0.96	90.8
9	0.5	1	7.5	5.69	46.8
10	1.5	1	2.5	0	~100
11	0.5	1	2.5	1.11	67.7
12	1	1.5	2.5	0	~100
13	1.5	1.5	5	0	~100
14	0.5	2	7.5	3.43	67.6
15	0.5	2	2.5	0.25	92.8

**Table 3:** EDX elemental composition of uncalcined LDH.

Element	Weight%	Atomic%	Mg/Al Ratio
C	5.56	7.9	-
N	5.27	6.4	-
O	64.57	68.9	-
Mg	18.55	13.0	3.4
Al	6.05	3.8	
Totals	100.00	-	-

**Table 4:** RSM modeling parameters for the RS and TS models.

Models	Significance Values for the Model Terms									
	Model	A: L:T Ratio	B: Dosage	C: SeCN^−^	AB	AC	BC	A^2^	B^2^	C^2^
RS	<0.0001	<0.0001	0.0002	<0.0001	0.021	<0.0001	0.0187	0.0115	--	--
TS	<0.0001	<0.0001	<0.0001	<0.0001	0.001	0.0017	0.0035	0.0468	0.0512	--

**Table 5:** Residual SeO_4_^2−^ (RS) and total selenium removal (TS) model fitting for selenocyanate treatment.

Statistic	RS Model	TS Model
R^2^	0.9868	0.9917
Adjusted R^2^	0.9736	0.9806
Predicted R^2^	0.9265	0.9188
Adequate Precision	29.99	34.46

**Table 6:** The constraints employed for RSM modeling for the photocatalysis work.

Name	Goal	Lower Limit	Upper Limit
A: LDH:TiO_2_	within range	0.5	1.5
B:Dose (g/L)	Reduce	1	2
C: Selenocyanate (mg/L)	7.5	2.5	7.5
Selenate remaining (mg/L)	reduce	0	5.7
Removal of selenate (percentage)	increase	47	100

## Data Availability

Data is contained within the article.
